# Supplementary figures and images for: BitterMatch: recommendation systems for matching molecules with bitter taste receptors
Source: J Cheminform. 2022 Jul 7;14:45. doi: 10.1186/s13321-022-00612-9 (PMC9261901; doi:10.1186/s13321-022-00612-9)

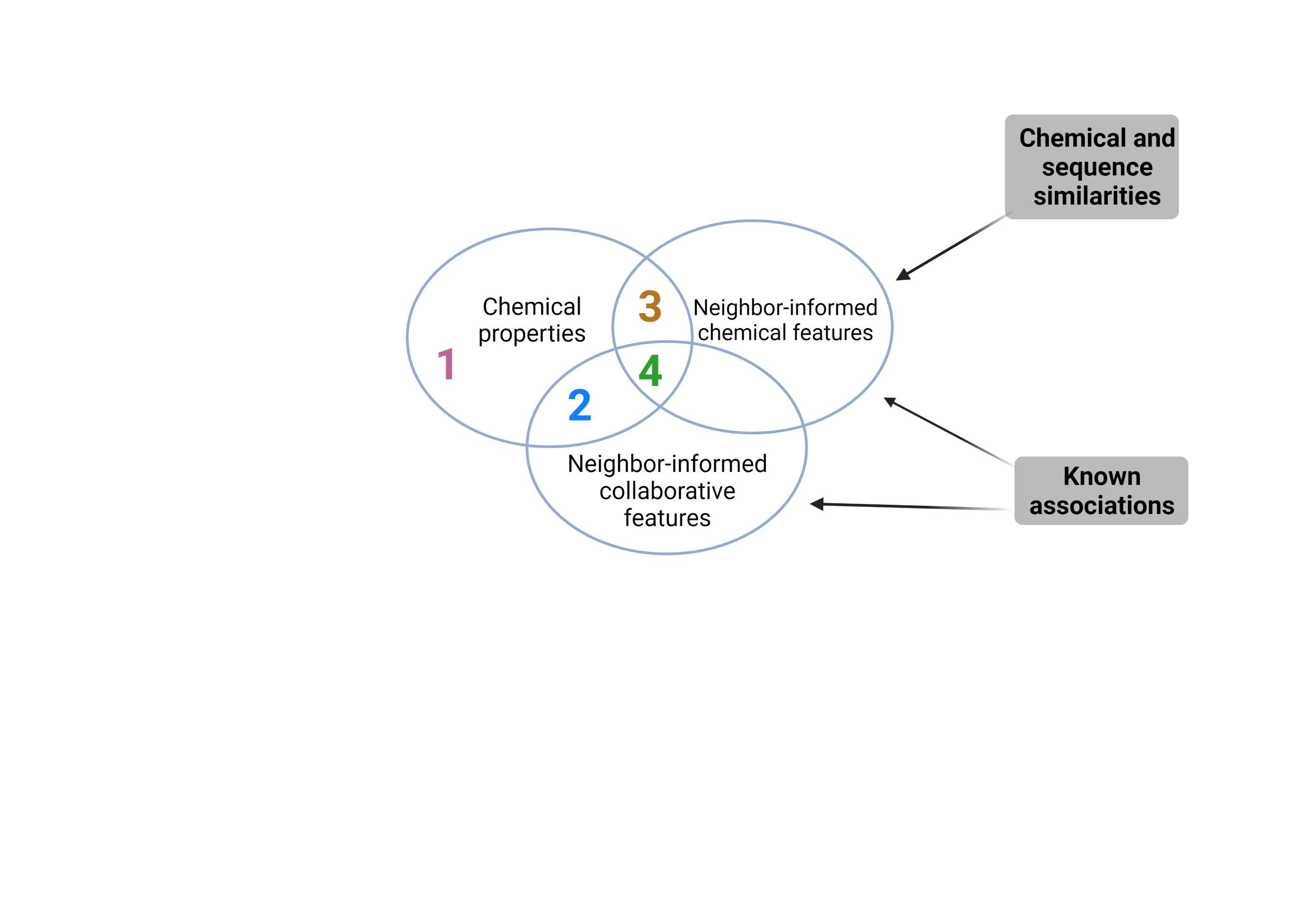

Supplement: Supplementary file 1 — Additional file 1: Figure S1. BitterMatch sub-models. A Venn diagram describing the four examined BitterMatch sub-models. Model (1) includes only chemical properties, model (2) includes chemical properties and neighbor-informed collaborative features, model (3) includes chemical properties and neighbor-informed chemical features, model (4) is the augmented one and it includes chemical properties, neighbor-informed collaborative features and neighbor-informed chemical features. Neighbor-informed collaborative features are computed directly from the known associations that were also used to calculate collaborative similarities. However, neighbor-informed chemical features are computed from the known associations and chemical and sequence similarities. [file 13321_2022_612_MOESM1_ESM.png]

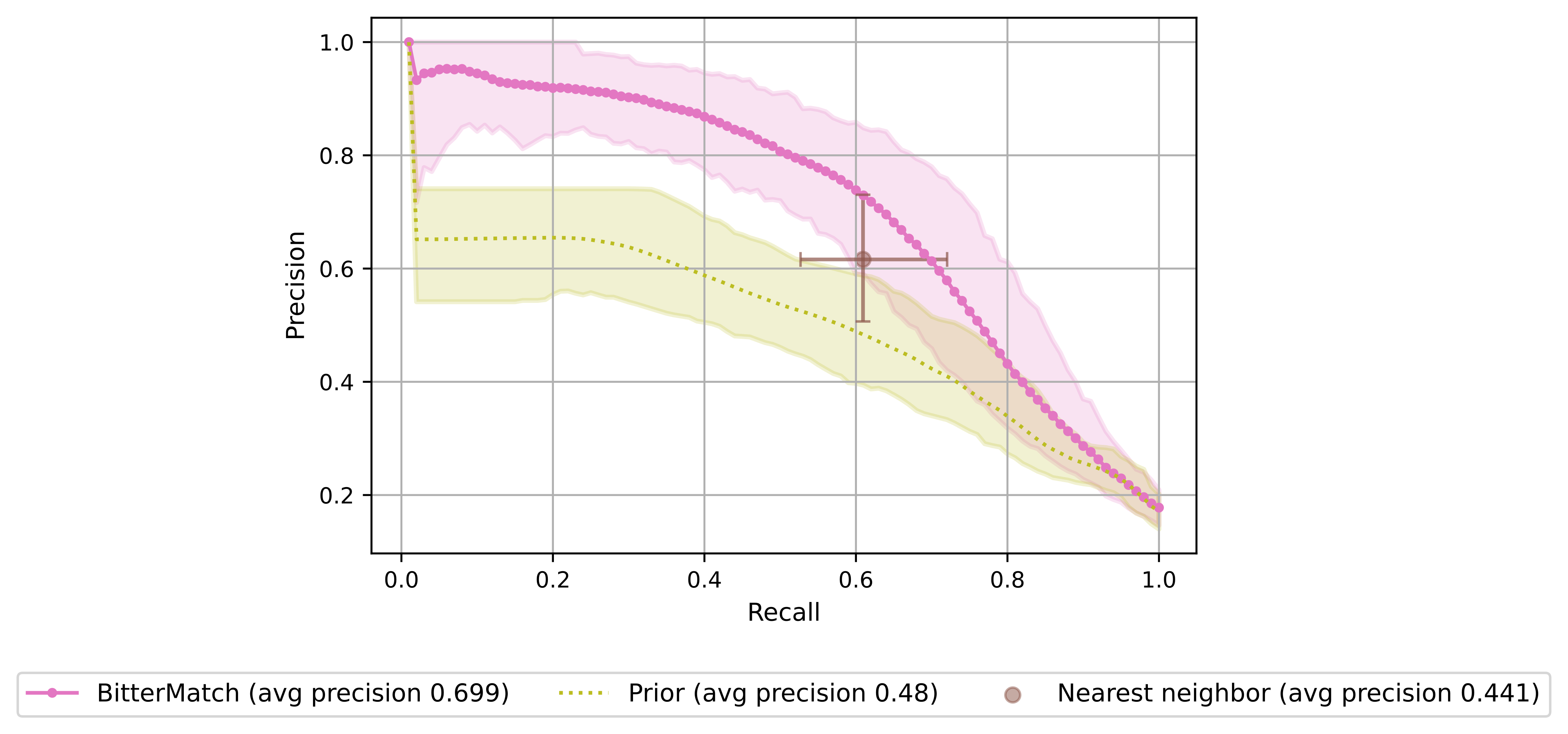

Supplement: Supplementary file 3 — Additional file 3: Figure S3. Prediction intervals for new ligands scenario. Precision-recall curves for the adapted BitterMatch model, a prior model and a nearest neighbor model (idenctivcal to Figure 4A). 95% Bootstrap prediction intervals are shown for each model. [file 13321_2022_612_MOESM3_ESM.tiff]

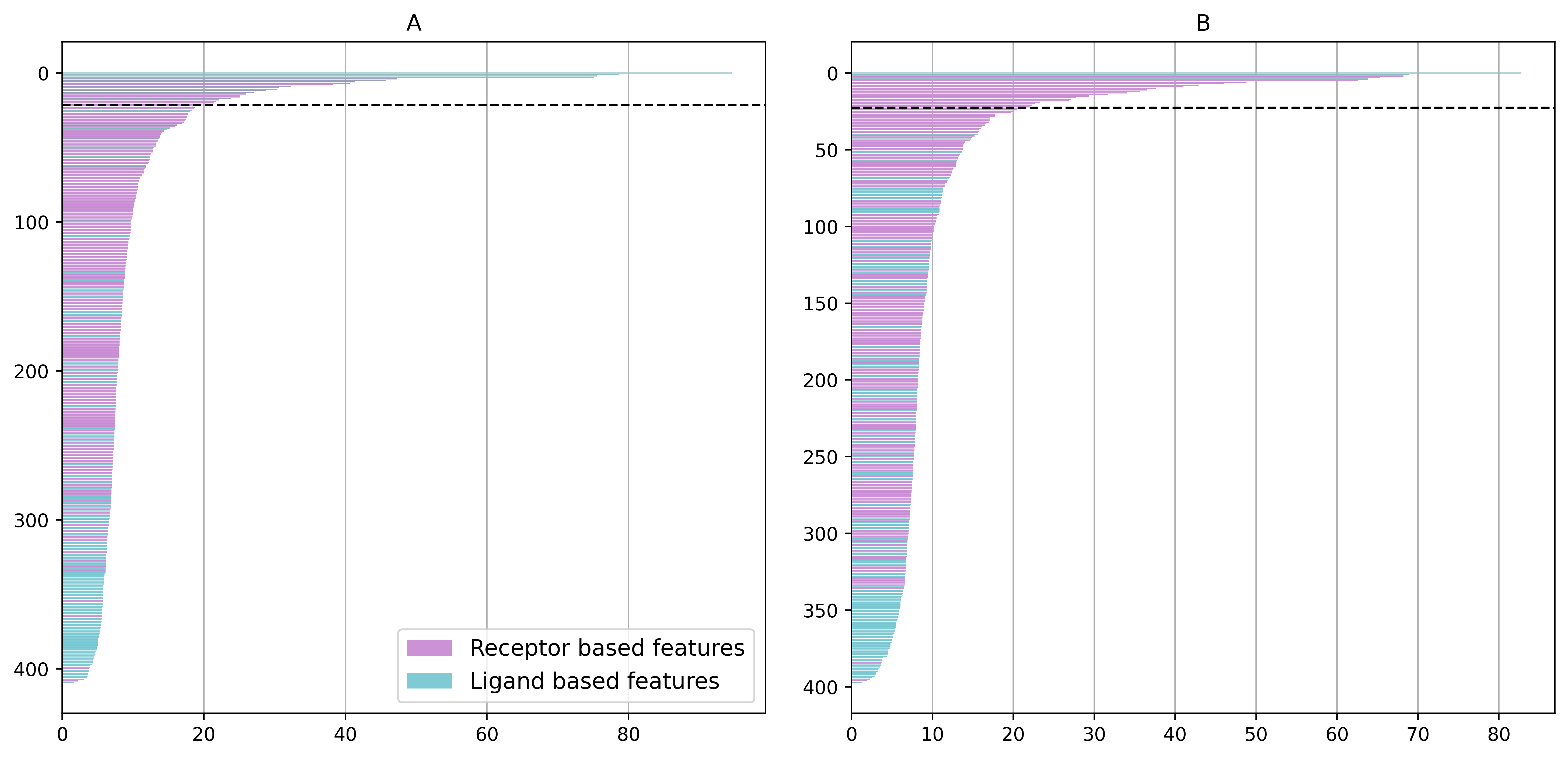

Supplement: Supplementary file 4 — Additional file 4: Figure S4. Feature importance for new ligands Features related to ligand similarities are shown in blue, receptor properties are shown in purple. (A) Feature importance for all the features in model 3. The black horizontal dashed line corresponds to feature importance of 21.5, features above this threshold are shown in detail in Fig. 6 (B) Feature importance for all the features in the "new ligands" model. The black horizontal dashed line corresponds to feature importance of 22.7. [file 13321_2022_612_MOESM4_ESM.tiff]

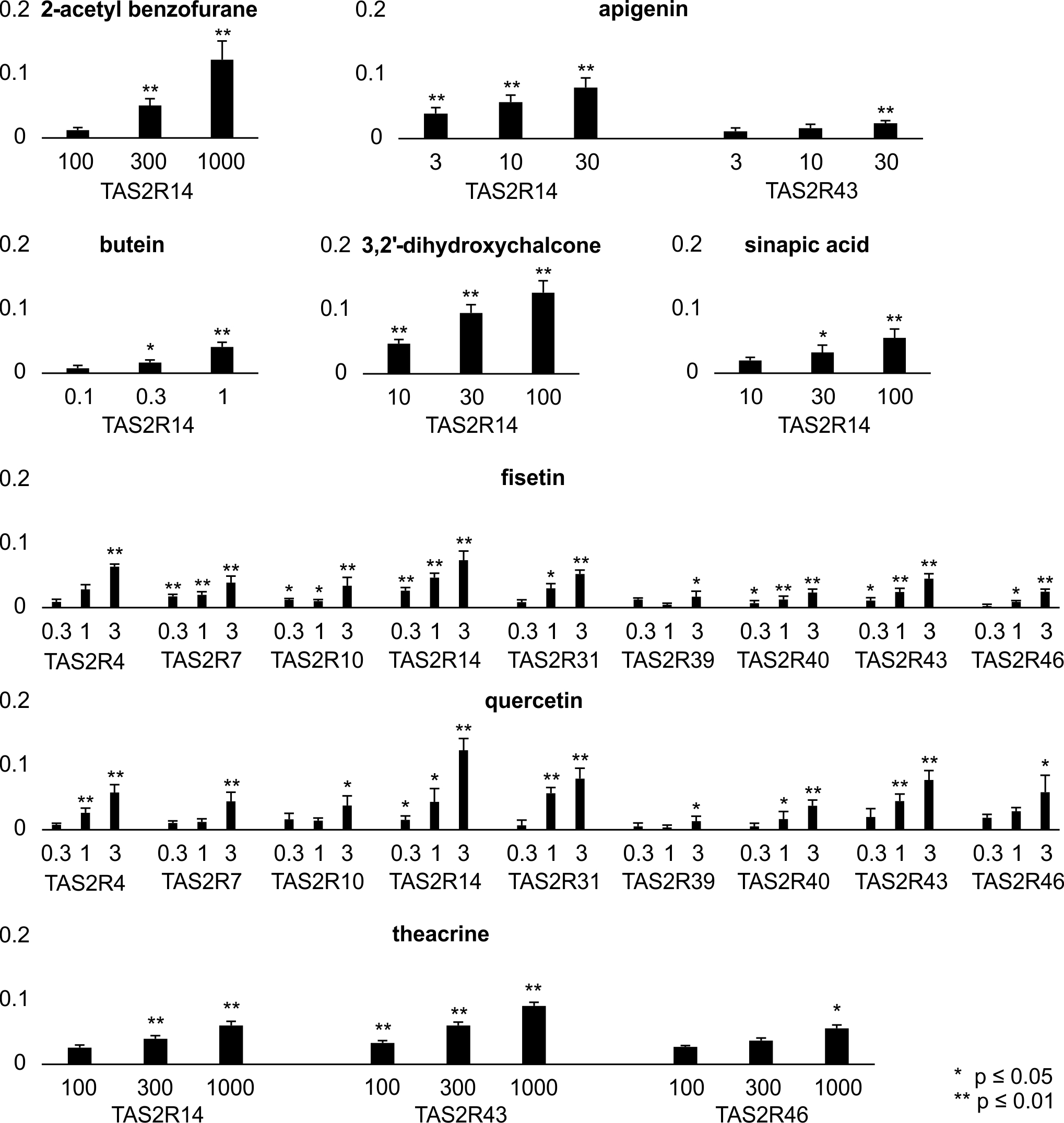

Supplement: Supplementary file 5 — Additional file 5: Figure S5. Confirmation of TAS2R screening results by functional experiments. The cDNA of the 9 TAS2Rs showing responses to the predicted bitter substances were expressed in HEK 293T-Ga16gust44 cells and challenged with 3 concentration of the corresponding newly identified agonists. The relative changes of fluorescence (ΔF/F) are provided on the y-axes of the bar graphs for each substance. Below the bars the concentrations used for the confirmatory experiments in µM are depicted together with the corresponding receptor symbols. The substance names are printed in bold. Statistically significant activations (Student’s t-test) are indicated by asterisks (see bottom right of figure for significance levels). [file 13321_2022_612_MOESM5_ESM.jpg]
