# Supplementary material for: BitterMatch: recommendation systems for matching molecules with bitter taste receptors
Source: J Cheminform. 2022 Jul 7;14:45. doi: 10.1186/s13321-022-00612-9 (PMC9261901; doi:10.1186/s13321-022-00612-9)
Supplement: Supplementary file 7 — Additional file 7: Table S1. Substances used for experimental testing of prospective predictions and their maximal employed concentrations. Table S2. XGBoost hyper-parameters. To avoid overfitting 1000 trees were used accordingly the following hyper-parameters were adjusted. The rest of the parameters were set to their default values. Table S3. Prospective prediction results per ligand. 1–represents an activation of the receptor that was confirmed experimentally and blank space means no activation was detected. *-positive activation that was detected in another publication but not in our in-vitro experiment. green–TP, red-FN, blue-FP and white-TN [file 13321_2022_612_MOESM7_ESM.docx]

**Table S1.** **Substances used for experimental testing of prospective predictions and their maximal employed concentrations.**

| **Substance** | **Max. concentration used for screening** | **Substance** | **Max. concentration used for screening** |
| --- | --- | --- | --- |
| 2-acetyl benzofurane | 1000 µM | Fisetin | 3 µM |
| Apigenin | 30 µM | Quercetin | 3 µM |
| Butein | 1 µM | Sinapic acid | 100 µM |
| 3,2’-dihydroxychalcone | 100 µM | Theacrine | 1000 µM |

**Table S2- XGBoost hyper-parameters.** To avoid overfitting 1000 trees were used accordingly the following hyper-parameters were adjusted. The rest of the parameters were set to their default values.

| **Parameter** | **Value** |
| --- | --- |
| Number of estimators | 1000 |
| Learning rate | 0.001 |
| Maximal depth of a tree | 4 |
| Column sample by tree | 0.3 |
| $\gamma$ - minimum loss reduction for partition on a leaf of a node | 2 |
| Minimum sum of instance weight needed in a child | 0.45 |
| Subsample - ratio of the training instances used prior to growing trees | 0.7 |

**Table S3 - Prospective prediction results per ligand.** 1 –represents an activation of the receptor that was confirmed experimentally and blank space means no activation was detected. * - positive activation that was detected in another publication but not in our in-vitro experiment. 1–TP, 1-FN, blue- FP and White - TN

|  | 4 | 7 | 10 | 14 | 39 | 40 | 43 | 44 | 46 |
| --- | --- | --- | --- | --- | --- | --- | --- | --- | --- |
| Sinapic acid   |  |  |  | 1 |  |  |  |  |  |
| 2-Acetylbenzofuran   |  |  |  | 1 |  |  |  |  |  |
| Theacrine   |  |  |  | 1 |  |  | 1 |  | 1 |
| Fisetin   | 1 | 1 | 1 | 1 | 1 | 1 | 1 | 1 | 1 |
| Quercetin   | 1 | 1 | 1 | 1 | 1 | 1 | 1 | 1 | 1 |
| Butein   |  |  |  | 1 | 1* |  |  |  |  |
| 3,2′-Dihydroxychalcone   |  |  |  | 1 | 1* |  |  |  |  |
| Apigenin   |  |  |  | 1 | 1* |  | 1 |  |  |
| Mozambioside   |  |  |  |  |  |  | 1 |  | 1 |
| Bengalensol   |  |  |  |  |  |  | 1 |  | 1 |
| Cafestol   |  |  |  |  |  |  | 1 |  | 1 |
| Kahweol   |  |  |  |  |  |  | 1 |  | 1 |
